# To grab the stroma by the horns: From biology to cancer therapy with mesenchymal stem cells

**DOI:** 10.18632/oncotarget.1040

**Published:** 2013-05-31

**Authors:** Ilia A. Droujinine, Mark A. Eckert, Weian Zhao

**Affiliations:** ^1^ Department of Genetics, Harvard Medical School, Boston, MA; ^2^ Sue and Bill Gross Stem Cell Research Center, Chao Family Comprehensive Cancer Center, Department of Biomedical Engineering, and Department of Pharmaceutical Sciences, University of California, Irvine, CA

**Keywords:** mesenchymal stem cells, cell therapy, stroma, microenvironment, tumor associated fibroblasts

## Abstract

Mesenchymal stem or stromal cells (MSCs) are precursor cells that play important roles in tumorigenesis. MSCs are recruited to tumors from local and distant sources to form part of the tumor microenvironment. MSCs influence tumor progression by interacting with cancer cells, endothelial cells, immune cells, and cancer stem cells, in a context-dependent network. This review aims to synthesize this emerging yet controversial field to identify key questions regarding the mechanisms of MSC mobilization and survival in blood; homing to tumors, metastases, and premetastatic sites; spatiotemporal organization and differentiation; and interaction with immune cells and cancer stem cells. Understanding the fundamental biology underlying mesenchymal stem cell and tumor interactions has the potential to inform our knowledge of cancer initiation and progression as well as lead to novel therapeutics for cancer. Furthermore, knowledge of endogenous mechanisms can be used to “program” exogenous MSCs for targeted chemotherapeutic delivery to tumors and metastases. Emerging studies will provide crucial insight into the mechanisms of tumor interactions with the whole organism including MSCs.

## 1. INTRODUCTION

Early molecular genetic studies on tumors primarily focused on cell-autonomous mechanisms of tumor progression. For instance, both growth and invasion were understood in terms of intracellular signaling pathways, regulation of cell cycle progression, apoptosis, and cytoskeletal dynamics (1). However, it has become increasingly evident that tumor cells non-cell autonomously interact with both their local microenvironment and the entire organism (2).

At the most basic level, tumor cells interact with their surroundings: the microenvironment or stroma composed of tumor promoting and opposing cells, soluble molecules, and extracellular matrix components (2). Tumor-associated cells include heterogeneous fibroblasts, neutrophils, macrophages, lymphocytes, endothelial cells, and nerve cells, among others. The extracellular matrix, in collaboration with soluble factors, provides signals for the modulation of cell cycle progression, apoptosis and migration of both tumors and associated stromal cells (3). Unlike the classical conception of a “disordered” tumor, these components form an intricate network of signaling and crosstalk, with subdivisions of processes and components (2).

Recent evidence indicates that non-hematopoietic stromal cells can originate from mesenchymal stem or stromal cells (MSCs), and MSCs themselves form a critical part of the tumor stroma. MSC are adult, non-hematopoietic multipotent stem cells that can be isolated from fat and bone marrow, among other tissues. MSCs are traditionally characterized by their plastic adherence, tri-lineage differentiation to adipocytes, chrondrocytes, and osteoblasts, expression of characteristic surface marker proteins (e.g., CD44, CD90, and CD106) (4). Local tissue-resident MSCs are important players in tissue homeostasis that are activated to proliferate and differentiate during tissue remodeling and inflammation. Under normal conditions, they may be associated with the local vasculature or other cells types (5). In many cases, however, MSCs are recruited from more distant organs to tumor and injury sites, including both the bone marrow (BM) and adipose tissue (6,7).

In recent years, there has been considerable interest in the use of MSCs as trophic vehicles for delivery of drugs, proteins, and other therapeutic agents specifically to tumors due to their lack of immune rejection and natural and specific ability to home to and integrate into tumors. The excellent phenotypic stability of MSCs in cell culture has facilitated their application in these technologies. In addition, MSCs may be easily modified, both genetically and non-genetically, for drug delivery, enhanced and more specific homing, and single-cell niche visualization—all with minimal impact on the MSC phenotype (8).

Here, we evaluate the role of both endogenous and exogenous MSCs and in some cases, MSC-like cells, as critical players in tumor progression. By endogenous MSCs we refer to those MSCs that are recruited to cancers from within the body; in contrast, exogenous MSCs are those that are cultured ex vivo before delivery to an experimental subject or patient. We emphasize that the process of MSC mobilization into the systemic circulation, survival in the blood, recruitment to tumors, differentiation, and distribution within tumors critically regulates tumor progression. The pleiotropic effects of MSCs include roles in regulating cancer stem cells, tumor proliferation, migration, immune cell recruitment and function, and angiogenesis. Moreover, we find extensive evidence that MSCs can be used as potent and safe tumor tropic vehicles for genetically engineered and conventional drug delivery to tumors. Together, these examples emphasize that the basic biology of tumor-associated cells can be engineered for both translation to the clinic and as experimental tools for investigation of regulatory interactions within the tumor. We hope that this article will both serve as a useful synthesis of the field as well as a source of intriguing open questions to be addressed in the future.

## 2. MSCS CONTRIBUTE TO THE TUMOR STROMA

**Figure 1:**
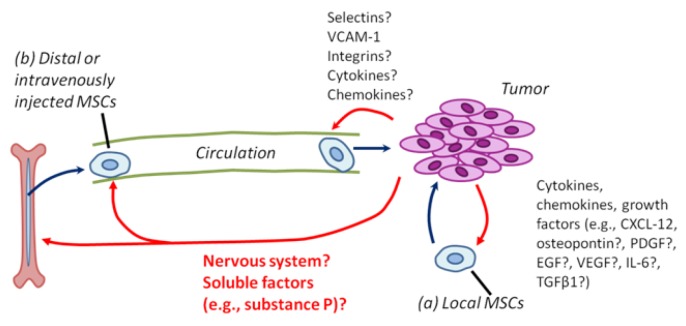
MSCs are recruited to tumors from local and distant (systemic) sources Red arrows show regulatory interactions of tumor cells on MSC biology; dark blue arrows show cell movements and action of MSCs on tumor cells. (a) Although the precise mechanisms remain to be determined, local (tissue-resident) MSCs can be recruited to tumors via tumor-derived cytokines, chemokines, and growth factors. (b) In addition, MSCs can be derived from distant (systemic) sources, including the bone marrow, adipose tissue, and possibly other organs. The signals mediating MSC mobilization to blood, survival in blood, and the attraction of MSCs to tumors are not known.

As tumors recruit many untransformed cells during cancer progression, many researchers naturally explored the contribution of endogenous stem cells to cancers. A growing body of evidence suggests that MSCs derived from both local (e.g., adipose tissue) and distant (e.g., bone marrow) sources contribute to the tumor stroma (Figure 1; Supplementary Table 1 and references therein). In tumors arising in the bone marrow or in metastases to the bone marrow, tumor cells may exploit endogenous hematopoietic stem cell niches for survival or proliferation (9,10). MSCs themselves have been proposed to form a critical part of the hematopoietic stem cell (HSC) niche (11), and tumor cells may compete for the HSC niche (9). Also, endogenous MSC-like cells (based on in vitro morphology and cell surface marker profiling) have been isolated from the bone marrow of chronic myeloid leukemia patients (12).

In addition to the bone marrow, MSCs are present in other tissues and may be recruited by tumor cells to form part of the microenvironment. Using ex vivo cultured MSCs as a model for endogenous local MSCs, several groups have shown important contributions of MSCs to the tumor stroma. For instance, in a rodent experimental glioma model, exogenous MSCs injected into the tumor were found to closely follow cancer invasive extensions and microsatellites (13), and human MSCs transplanted locally into the tumor were able to migrate to and survive in the cancer mass (14). These results suggest that molecular interactive cues might exist between glioma cells and MSCs (13). In addition, locally injected exogenous MSCs preferentially localized to experimental intraperitoneal tumors (15). Significantly, real time non-invasive data with exogenous luciferase-expressing Human MSCs (hMSCs) indicated that hMSCs incorporate into tumors where they may contribute to the tumor microenvironment (15,16).

Endogenous MSC-like cells populate the uninjured skin and other organs, and contribute to wounded eye and skin epithelium, injury-induced arterial intimal hyperplasia, and ischemic myocardium (17–21). This raises the question of to what extent endogenous MSCs can integrate into tumors, which several studies have directly addressed. In several elegant studies, immunodeficient mice were sublethally irradiated and transplanted with green fluorescent protein (GFP) labeled bone marrow to label bone-marrow derived cells, including MSCs. It was found that bone marrow-derived myofibroblasts were present in pancreatic cancers and that the proportion of bone marrow-derived cells increased as the tumor progressed (22,23). Moreover, in a similar study using GFP-labeled bone marrow, about half of ovarian tumor stromal cells were derived from the bone marrow; in this case accounting for 20% of the myofibroblasts and pericytes and 70% of the fibroblasts. A sizeable proportion of stromal cells originated from MSCs and more than 75% of MSC-derived tumor stromal cells were fibroblast-like, while up to 10% were myofibroblasts (6). This observation extends to inflammatory models of gastric cancer, where 20% of all cancer myofibroblasts were bone marrow derived (24). Interestingly, myofibroblast-like cells increased in number in the BM of tumor-containing versus tumor-free mice, with in vitro evidence suggesting that MSCs can give rise to myofibroblasts (24). Intriguingly, there is preliminary evidence of bone marrow contribution to the stroma of human tumors. Worthley et al. performed an analysis of tumor tissue specimens obtained from female allogenic bone marrow transplant patients that had received bone marrow from male donors and found myofibroblasts in gastrointestinal tumors that possessed Y-chromosomes (25).

In addition to the bone marrow, local resident MSC-like cells may also contribute to the tumor stroma. Using transplantation of GFP-positive bone marrow, Kidd et al found that approximately half of the tumor stromal cells were of non-bone marrow origin (6). In contrast, almost all endothelial cells and the majority of pericytes were derived from cells outside the bone marrow. Furthermore, transplantation of fluorescently labeled adipose tissue as a source of stromal cells adjacent to the tumor revealed a significant contribution of adipose-derived cells to the tumor stroma, particularly pericytes. Although the exact nature of the cells giving rise to tumor stromal elements is not clear, these results indicate that local and distant stromal sources may contribute to the tumor stroma and that their relative contributions to different stromal elements may differ (6). In support of this hypothesis, a recent study found that endogenous local MSC-like adipose stromal cells mobilize and engraft to the tumor in an obese mouse model of cancer, emphasizing the importance of systemic communication between organs and cancer progression (7).

Intriguingly, tumor cells themselves could be a source of cells with MSC-like characteristics. Battula et al (2010) demonstrated that immortalized human mammary epithelial cells induced to undergo epithelial-mesenchymal transition (EMT) in vitro by Twist, Snail, or transforming growth factor β (TGFβ) overexpression recapitulated many of the features of MSCs. Namely, they expressed MSC surface markers and gained the ability to differentiate to chrondrocytes, osteoblasts and adipocytes (26). This raises the interesting possibility that tumor cells may give rise to their own stroma through transdifferentiation. Interestingly, recent work showed differentiation of glioblastoma stem cells to pericytes (which may resemble MSCs), dependent on TGF-β (27).

Finally, numerous studies have demonstrated that ex vivo cultured “exogenous” MSCs (but not their differentiated progeny) efficiently home to tumors and metastases after intravenous injection (Supplementary Table 1 and references therein). In an uninjured mouse, exogenous intravenously-injected MSCs rapidly accumulate within lungs and are cleared to other organs such as the liver within days. Moreover, MSCs are not detectable in the mouse body after 7 days (16). This pattern of accumulation followed by clearance within days is markedly similar to that of non-reactive acrylic microspheres, suggesting that passive accumulation dominates the biodistribution of MSCs under normal physiological conditions (27). A significant body of work has firmly established that exogenously cultured MSCs are capable of selectively homing to and surviving in a variety of pre-established solid tumors (breast, colon, melanoma, and others) while being excluded from normal tissues. The evidence for this has been obtained using MSC-specific PCR, immunohistochemistry (IHC) (29–31) and non-invasive, real-time bioluminescence imaging (16,32,33), nuclear magnetic resonance imaging (MRI) (34), positron emission tomography (PET) (35), and radionuclide imaging (35). In addition to systemic targeting of large solid tumors, MSCs (but not differentiated fibroblasts) have been shown to home to and be retained in metastases and other small tumors, while not accumulating in normal tissues. This was demonstrated using methods including immunohistochemistry (29,36–38), and bioluminescence imaging (16,32) in cancers ranging from breast to prostate to melanomas. These studies have provided a model for studying MSC homing to tumors and emphasized the possibility of using MSCs as drug delivery vehicles to tumors.

## 3. MECHANISMS OF MSC RECRUITMENT AND HOMING TO TUMORS

Mobilization from the bone marrow and other organs represents the first key step in MSC homing to tumors. There is evidence that endogenous MSCs can mobilize from the BM and other tissues to the peripheral blood under both normoxia and hypoxia, inflammation, and injury conditions (18–20). Moreover, this process is tightly regulated, with endogenous MSC-like cells only being isolated and cultured from the blood of mice subjected to femoral artery injury, but not from the blood of uninjured mice (19). Similarly, CD29-positive MSC-like cells could be isolated and cultured from blood of mice with burn-injured corneas, but not from uninjured mice (18). Although multiple studies have found that the bone marrow plays key roles in contributing to the tumor stroma (Supplementary Table 1 and references therein), major unresolved questions remain regarding the mobilization of MSCs from the bone marrow and homing to tumors (6,20,24).

Inflammatory cytokines have been suggested to mediate the mobilization of MSCs from their tissues of origin, as their concentration increases during injury and other disease processes, concomitant with an increase in circulating MSCs (18–20). In response to cornea burn injuries, it was found that the 11-amino acid neuropeptide, substance P, was elevated in the blood of injured animals and was sufficient to mobilize endogenous MSCs to the blood (18). In addition, other systemic signals may also be critical in mobilizing MSCs to blood. For example, BM MSC have been shown to respond to sympathetic nervous system (SNS) signals to regulate hematopoietic stem cells (11). Since SNS signals have been observed to influence tumor progression (39), the possibility of regulating MSC mobilization through the SNS in the context of tumors is an intriguing area of research that has not yet been explored. Despite some key observations, the initial key steps in MSC mobilization and intravasation into the blood stream remain largely unexplained.

**Figure 2:**
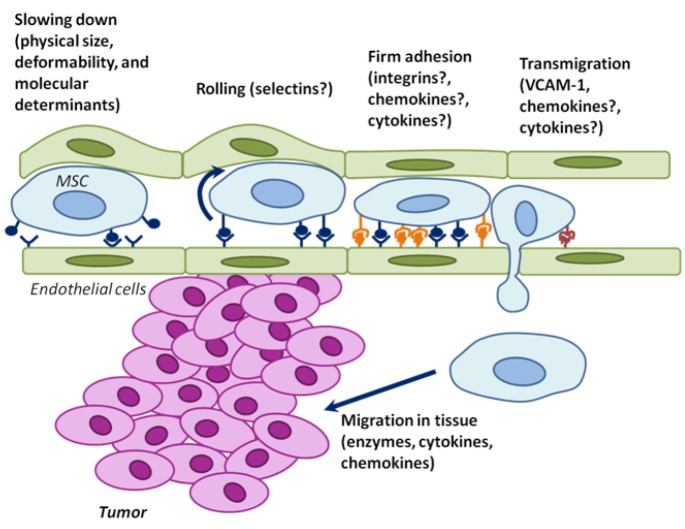
The physical parameters and cell surface molecules of MSCs cooperate to induce active homing of MSCs to tumors Endothelial cells are green; MSCs are blue; and tumor cells are pink. Although the mechanisms of MSC homing are poorly understood, they likely involve partially overlapping steps of deceleration in the blood flow (which may be partially physical – here depicted as bulging endothelial cells), rolling, adhesion, transmigration through the endothelium, and migration into surrounding tissues. The possible molecular determinants are indicated. The elucidation of the MSC homing mechanism to tumors will facilitate the development of drugs for inhibition of this process (endogenous MSCs), or for understanding how ex vivo cultured MSCs can be engineered to be used as effective and specific drug delivery vehicles.

Following mobilization from distant sources into the systemic vascular system, MSCs must then extravasate and engraft at tumors. Much work dedicated to understanding immune cell homing has established that the steps of interaction between cells in the circulation and endothelial cells (ECs) include capture, rolling, activation, arrest, adhesion strengthening, crawling, transendothelial migration and establishment within target tissues. Selectins, selectin ligands, integrins-immunoglobulin superfamily receptors (e.g., vascular cell adhesion molecule-1 – VCAM-1), chemokines and their receptors, and other molecules are involved in this process (40). Despite controversy over the exact mechanism (Figure 2), there is strong evidence that MSCs have the capacity to home to sites of inflammation. Moreover, MSC adhesion to the endothelium is greatly enhanced by activation of the endothelium with pro-inflammatory cytokines such as tumor necrosis factor α (TNFα) (41). As many cancers induce significant inflammatory responses, this suggests a possible mechanism regulating the tropism of MSCs for solid tumors (42). Recent data with inhibitors to chemokine (C-X-C motif) ligand 12/stromal cell-derived factor 1 (CXCL12/SDF-1) receptor CXCR4 and TGFβ receptor suggest that endogenous MSC homing to tumors, differentiation to myofibroblasts, and/or survival require CXCR4 (24). In addition several studies have suggested that tumor-derived platelet-derived growth factor (PDGF), epidermal growth factor (EGF), vascular endothelial growth factor (VEGF), and interleukin-6 (IL-6) may be required for localization, integration and/or survival in tumors (43–45).

The role of cytokines and chemokines in the context of MSCs has also been examined and found to induce survival and engraftment and promote enhanced slow crawling (46), adhesion (41,46), spreading on the endothelium (46), transmigration (46,47), and chemotaxis (48,49). A recent study compared the transmigration of MSCs, leukocytes, and cancer cells. In contrast to leukocytes, but similar to cancer cells, MSC transmigration does not involve lamellopodia or invadosomes, but instead involves “blebbing” of their membranes. Although the regulation and molecular mechanisms remain to be characterized, blebbing and VCAM-1 have been proposed to be important for establishing transmigration interactions with the endothelium (47).

The BBB is a well-regulated barrier to cell trafficking to the brain that normally prevents extravasation of immune and other cells to the brain parenchyma (50). Interestingly, exogenous MSCs appear to be capable of crossing the blood-brain barrier (BBB) and contribute to experimental gliomas when administered via the carotid artery(13). At 1 hour following injection, MSCs were clustered near blood vessels, suggesting entry of the MSCs through the endothelial cells. By 3-4 days post injection, human MSCs were dispersed throughout the tumor, but were not present within normal adjacent and distant brain tissue. Similar results were obtained in a similar glioma model (31), and robust MSC transmigration of in vitro models of the BBB have been observed (51). As MSCs have been additionally utilized as therapeutics for a variety of central nervous system disorders, (47, 51) understanding the mechanisms by which they bypass the BBB has clear clinical implications for both cancer and other disorders.

It is likely that MSC homing to tumors involves interplay between active recruitment via chemokines and inflammatory processes and passive entrapment in the vasculature. Exogenously delivered MSCs are physically and molecularly non-specifically entrapped in sites with small blood vessels, including “filtering organs” like the lungs, liver, and spleen (52,53). Intriguingly, use of a vasodilator concomitant with injection of exogenous MSCs led to a markedly altered biodistribution of MSCs, with a significant decrease in entrapment in the lungs (52). Moreover, treatment of exogenous MSCs with an integrin α4 blocking antibody results in entrapment of significantly fewer cells in the lung following intravenous injection (54). Thus, we reason that the physical parameters of MSCs collaborate with molecular determinants to ultimately dictate homing and engraftment. Unraveling the relative contributions of molecular signals and physical parameters in MSC homing to tumors will reveal both new biology and novel targets for therapeutic intervention.

## 4. MSC ORGANIZATION AND DIFFERENTIATION WITHIN TUMORS

**Figure 3:**
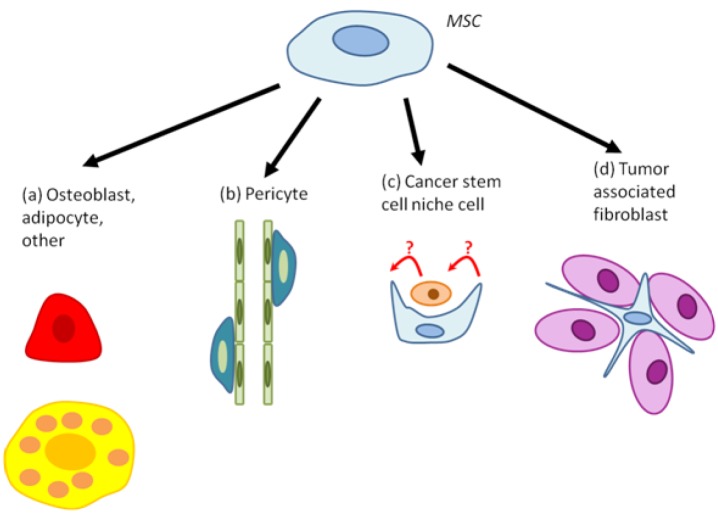
MSC differentiation within tumors MSCs have been found to differentiate to (a) osteoblasts (red), adipocytes (yellow), or other cell types; (b) pericytes; (c) cancer stem cell niche cells; and (d) tumor associated fibroblasts. The precise function of each of these cell types remains to be determined. Each step may be targeted for inhibition by therapies, based on their function in promoting or inhibiting tumor progression or initiation.

Although MSC tropism for tumors is now relatively well established, their ultimate fate - incorporation into tumors, differentiation, communication with cancer cells, other stromal cells, and cancer stem cells - in the tumor remains unclear. We have summarized the key findings in literature regarding MSC organization and differentiation within tumors in Figure 3 and Supplementary Table 2. Briefly, MSCs incorporate into tumors as single cells and clumps that may be associated with both necrotic regions and the vasculature. MSCs have been found to contribute to multiple cellular components of the stroma, including fibroblasts, periendothelial cells, adipocytes, and osteoblasts, among others. In particular, MSC-derived cells localized to lung metastases underwent osteogenic differentiation, while MSCs localized to subcutaneous tumors adopted an adipogenic fate. These results indicate that the lung and subcutaneous locations of tumors may have different microenvironmental influences on tumor and MSC fate (32). Interestingly, changing the microenvironment with irradiation of subcutaneous tumors was sufficient to alter intravenously injected mouse MSC distribution within it. Whereas MSCs were mostly associated with blood vessels in non-irradiated tumors, irradiation caused MSCs to become more localized to the tumor parenchyma, which could have important implications for therapy that utilizes MSCs as delivery vehicles (33). Furthermore, recent studies have suggested that MSCs may form a part of the cancer stem cell (CSC) niche (Figure 3c). Specifically, it was found that human MSCs transplanted into the bone marrow can expand the putative breast cancer CSC population via a cytokine loop involving IL6 and CXCL7 (45). Moreover, MSCs were able to home to the tumor, distribute throughout the stroma mostly as single cells, and closely associate with the putative CSCs.

Tumor MSC organization or differentiation is a regulated event that depends on the tumor context (e.g., see Klopp et al., 2007 and Wang et al., 2009) (32,33). Mechanistically, MSC organization, survival, and differentiation depends in part on CXCR4, TGF-β, Wnt5a, IL-6 signaling, supplied by myofibroblasts, which serve as niche cells for MSCs in tumors. TGF-β induced the differentiation of MSCs to myofibroblasts, in a CXCR4-dependent manner. Myofibroblasts expressed bone morphogenic protein 4 (BMP4), Wnt5a, and IL-6, and MSCs expressed the BMP inhibitor Gremlin-1 when in close proximity to myofibroblasts. Also, expression of the Wnt inhibitors DKK1 and Shh was dependent on co-culture of MSCs with myofibroblasts, suggesting a delicate signaling network between the two cell types (24). Understanding the functional relevance of MSCs in relationship to both their organization and differentiation will be essential for fully defining the roles of MSCs in cancer.

## 5. THE FUNCTION OF MSCS IN TUMORS

**Figure 4:**
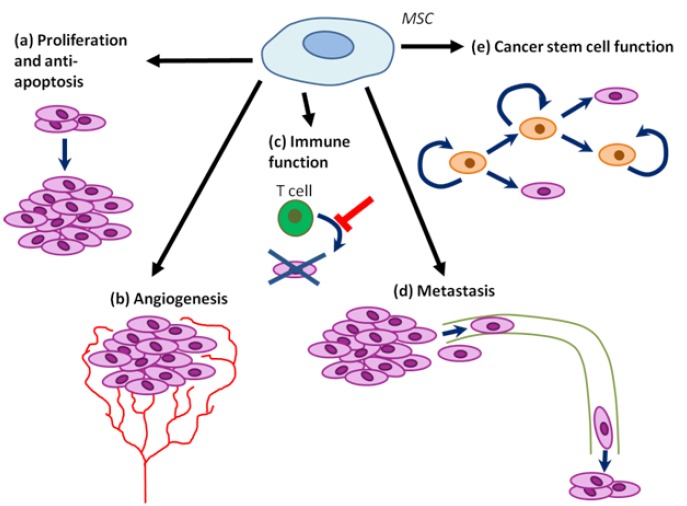
MSC tumor progression promoting functions (a) MSCs may promote tumor cell proliferation and inhibit cell death. (b) MSCs may promote angiogenesis within tumors. (c) MSCs may inhibit some immune functions, while promoting others. (d) MSCs may promote tumor metastasis, possibly at multiple steps (from initial dissemination to formation of novel niches in distal tissues). (e) MSCs may regulate cancer stem cell self-renewal and differentiation.

MSCs regulate the tumor phenotype by affecting the initiation and growth of tumors, angiogenesis, metastasis, immune system function and cancer stem cell function, in a context-dependent manner which we summarized in details in Figure 4 and Supplementary Table 3. In addition, endogenous bone marrow and adipose tissue-derived MSCs may directly contribute to the tumor stroma. As alluded to above, MSCs derived from the bone marrow may give rise to tumor myofibroblasts and promote the growth of gastric tumors (24). In addition, both adipose and bone marrow MSC sources may contribute to the tumor stroma, with the bone marrow contributing to the tumor-associated fibroblasts and adipose tissue contributing to the vascular and fibrovascular stroma (6).

MSCs are known to secrete a number of anti-apoptotic and pro-proliferative cytokines and growth factors that may act directly on tumor cells (Da Silva Meirelles et al., 2008; Wu et al., 2010). For example, MSCs promote tumor chemotherapy resistance via secretion of omega-3 and oxo family fatty acids (55). Moreover, MSCs differentiate to tumor pericytes and MSC-secreted VEGF and other pro-angiogenenic molecules induce tumor angiogenesis (7,43). Also, tumor-distal or resident MSCs enhance cancer cell motility, extravasation to secondary sites (56), and possibly also intravasation to and survival in blood. Tumor associated fibroblast (TAF) secreted CXCL-12 (SDF-1) was found to directly promote the growth of CXCR4 expressing tumors in vivo and in vitro (57). A large proportion of MSCs contribute to the TAF population, raising the possibility of MSC involvement in activation of this pathway (6). The exciting possibility that MSCs may contribute to the pre-metastatic niche remains to be explored (58). In addition, MSC-like cells contribute to the hematopoietic niche and thus may contribute to bone marrow metastases (59).

MSCs also interact with immune cells in tumors, inhibiting or promoting inflammation depending on the context. While MSCs may inhibit leukocyte proliferation in solid tumors (60), MSCs may promote proliferation of leukemia cells (12). MSCs decrease the proliferation and differentiation of B cells via prostaglandin E2 (PGE2). Moreover, MSCs suppress activation of helper T cells via PGE2, indoleamine 2,3-dioxygenase (IDO), TGFβ, hepatocyte growth factor (HGF), nitric oxide (NO), and haem oxygenase 1, while the activation of cytotoxic T cells may be inhibited but regulatory T cells promoted by human leukocyte antigen G5 (HLA-G5) (61). Also, MSC-derived IDO, PGE2, and TGFβ decrease the activation and killing activity of natural killer cells, while IL-6, IL-10, TGFβ, PGE2, HGF, and macrophage colony-stimulating factor (M-CSF) inhibit the differentiation and function of dendritic cells. IL-6 also negatively regulates neutrophils (62,63).

Also, MSCs form part of the cancer stem cell niche and modulate their function, at least in part via BMP, PGE2, and β-catenin signaling (45,64). Two recent reports suggested that human bone marrow (45)- or tumor-derived (65) MSC-like cells (with differentiation capacity, immunophenotype, and colony formation ability similar to MSCs) enhance the growth of tumors by positively regulating the proliferation and/or self-renewal of ALDH+ CSCs. In addition, MSCs increased the mammosphere formation capacity of tumor cell lines in vitro (65) and the proportion of ALDH+ CSC-like cells in vivo (45). Similar results were obtained by McLean et al, who demonstrated that MSC-like cells isolated from human tumors induce the growth of tumors and the self-renewal of putative CSCs at least in part via BMP secretion (which has been previously shown to be involved in tumor progression) (65). In addition, Quante et al suggested that the MSC-derived myofibroblasts create a niche for stem-cell like MSCs. Molecules involved in the niche were likely BMP4, Wnt5a, IL-6, Gremlin-1, DKK, and Shh. This raises the question of whether similar molecules may be involved in the maintenance of the CSC niche (24). Intriguingly, a recent paper from the Weinberg laboratory found that a delicate crosstalk between MSCs and cancer cells plays roles in both inducing CSCs and regulating the phenotype of tumor-associated MSCs (64). Specifically, they found that secretion of IL-1 by tumor cells induces PGE2 secretion by MSCs; PGE2, in combination with upregulation of cytokines by MSCs, leads to activation of β-catenin signaling in cancer cells and subsequent formation of CSCs. This paper not only emphasizes the plastic nature of CSCs, but also the dynamic interactions between CSCs and MSCs in cancer progression and maintenance.

Surprisingly, Khakoo et al found that Kaposi sarcoma tumors in mice are growth inhibited when in the presence of MSCs (34). It thus appears that MSC functional contribution to tumor progression is highly context dependent, with this inherent variation compounded by inconsistencies in MSC preparation. Indeed, differences may arise from differences in tumor models, MSC preparations, and timing and concentration of MSC administration (66). Carefully defining the roles of MSCs in regulating the initiation versus progression of tumors in the context of any experimental design will be essential for understanding the results of any experiments. For example, some tumor cell lines growth in vivo is promoted by MSCs while others are not (within a single study). Also, another group observed that while high numbers of MSCs within tumors may promote tumor initiation, a low number of MSCs may have no or inhibitory effects, suggesting complex dosage effects in pro- and anti- initiation pathways which remain to be elucidated (67). In addition, Khakoo et al suggested a way in which tumor genetic context can influence the effect of MSCs on tumor progression. Inhibition of human Kaposi sarcoma cell growth was dependent on cell contact via E-cadherin and Akt inhibition (34). Thus, it would be of interest to conduct systematic loss and gain-of-function studies on endogenous MSCs and MSCs derived ex vivo, with different exogenous or endogenous tumor types (66). It is likely that tumor type, stage, and the genetic dependencies of the tumor all influence the exact roles of MSCs in regulating tumor progression or initiation.

Several studies suggest that MSCs promote tumor metastasis though secretion of SDF-1, CCL-5, and other chemokines. Tumor cells induced expression of CCL5 (regulated and normal T cell expressed and secreted; RANTES) in MSCs, which in turn increased tumor migration and metastasis in a paracrine and/or endocrine manner (56). In support of this hypothesis, one group found that neuroblastoma tumor cells can migrate towards MSCs in vitro and that this response was dependent on SDF1-CXCR4 signaling (68). Moreover, MSC-like cells have been proposed to contribute to the bone marrow hematopoietic niche and hence may promote the establishment of niches for metastatic cells (59). Thus, MSCs present in target tissues may induce migration of metastasizing tumor cells (5,59).

## 6. MSC USE IN CANCER THERAPY

**Figure 5:**
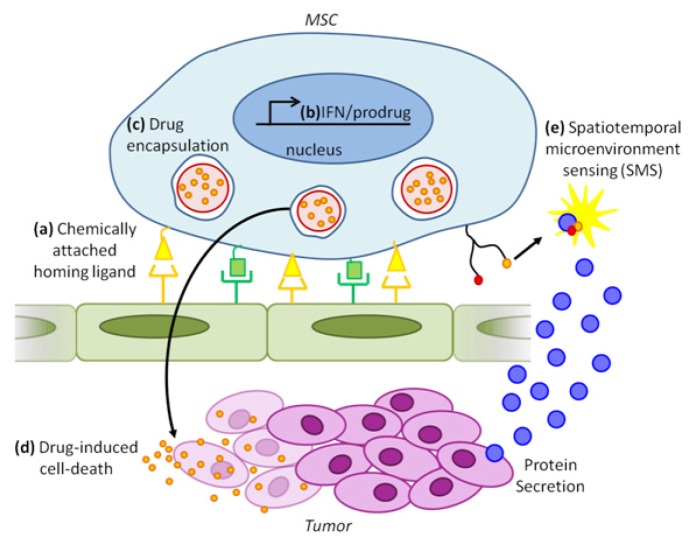
Combinatorial cancer therapy and detection using engineered exogenous MSCs (a) Cell surface chemistry using biotin-streptavidin conjugation or enzymatic modification can be used to attach multiple ligands (yellow and green) on the MSC surface. Different combinations of these ligands can be used to combinatorally target MSCs to specific tissues in the body (e.g., tumor). (b) Genetic engineering techniques may be used to express anti-tumorigenic molecules including interferons (IFN) and prodrug converting enzymes. (c) Drugs (small orange circles) can be encapsulated in micro or nanoparticles (large red circles), which in turn can be engulfed by MSCs. (d) Drug release from MSCs will cause tumor cell death. (e) Spatiotemporal micronenvironment sensing (SMS): Tumor microenvironment, intercellular signaling, and tumor progression may be imaged via molecular probes such as surface-conjugated FRET aptamers.

In recent years, there has been considerable interest in the use of MSCs as trophic vehicles for delivery of drugs, proteins, and other therapeutic agents specifically to tumors. The advantage of using MSCs for drug delivery versus using other tumor-tropic cells such as macrophages include the immunoprivileged status of MSCs, homing abilities and intratumoral distribution, availability, genotypic and phenotypic stability, expandability, and proven safety record in clinical trials (69,70). Given the possibility of MSC regulation of tumor function it would be desirable to have viable drug-containing MSCs with stable functional properties until integration, distribution and drug release within tumors; however, once this has occurred, MSCs should die in order to avoid effects that facilitate tumor progression. The safe use of MSCs to treat cancer or non-cancer diseases in patients that have undiagnosed, early-stage cancer requires understanding the fate and functions of MSCs in vivo and their interactions with tumors. Figure 5 and Supplementary Table 4 summarize the approaches in this field. To circumvent use of genetic engineering, incorporation of drug-laden nano/microparticles on the cell surface or intracellularly, has been done (Supplementary Table 4 and references therein).

## 7. CONCLUSIONS AND PERSPECTIVES

Endogenous and ex vivo cultured MSCs have been shown to specifically home to, integrate with, and survive in tumors. This property has successfully been used in experimental tumor therapies. Table 1 summarizes central questions remaining in the field and how they may be addressed with future work. Although we are beginning to understand how MSCs interact with tumors and how this can be exploited therapeutically, many questions and hurdles remain.

**Table 1:** Key Directions for Future Studies

Questions or problems	Suggested solutions and approaches
MSC-cancer interactions	
What distal tissues do MSCs mobilize from to contribute to tumors?	MSC labeling in different tissues (e.g., genetic via Cre recombinase) and tracking MSCs in tumors
What are the mechanisms of MSC mobilization to and survival in blood?	Identification of tumor-derived factors that mobilize MSCs from distal tissues Two-photon intravital microscopy (IVM) Function blocking MSC survival and recovery assays in blood MSC interaction with other blood cell types In vivo and in vitro RNAi screening using labeled MSCs
What are the cell subtypes that MSCs differentiate to and what are the roles of these cells?	Detailed in vivo lineage characterization after MSC labeling (e.g., genetic via Cre recombinase); progeny isolation and characterization or in situ characterization Depletion of MSC progeny via genetic, pharmacological, or other techniques; determine the effect on tumor progression
What are the mechanisms of MSC homing to, survival in, and organization (differentiation and localization) within tumors and normal organs?	In vitro flow chamber In vivo two-photon intravital microscopy (IVM) and MSC-surface aptamer sensors Function blocking by antibodies, RNAi, or pharmacology In vivo and in vitro screening using labeled MSCs
What are the mechanisms of MSC interaction with immune cells in cancers?	In vivo two-photon intravital microscopy (IVM) and MSC-surface aptamer sensors In vitro interaction studies of MSC with tumor and normal immune cells
What are the mechanisms of MSC interaction with cancer stem cells?	In vivo two-photon intravital microscopy (IVM) and MSC-surface aptamer sensors In vivo depletion of MSCs in tumors by genetic or pharmacological techniques, to determine effects on phenotype and proportion of cancer stem cells In vivo function blocking and proportion of cancer stem cells in tumors
MSC-based cancer therapeutics	
Design of therapies targeting tumor microenvironment components	Use of knowledge of mechanisms of endogenous MSC homing, integration, and function in tumors to design therapies for blockade of tumor and metastatic progression
Localized drug delivery to tumors	Use of non-genetically engineered MSCs for in vivo drug delivery (engulfed microparticles)
Integration of biology and therapy	Combinatorial modification of MSCs for enhanced homing (surface SLeX), single-cell tumor microenvironment imaging (surface aptamers), and multiple drug delivery (microparticles) Combinatorial therapy using MSCs and other therapies

Although both bone marrow, adipose, and local tissues are sources of tumor-associated MSCs, their relative contribution, kinetics, and regulation remain poorly understood. A solution is to label progeny of local and distant MSCs to follow their fate and dynamic behavior in the tumor. However, the absence of specific and reliable markers of MSCs has been a hurdle to the generation of reporter mice. Current studies have therefore been limited to the use of BMT from labeled mice. The development of MSC reporter mice would provide an elegant and revolutionary tool for investigation of MSCs in cancer.

To better understand the recruitment of MSCs to tumors, it will be important to study the mechanisms of MSC mobilization and survival in the circulation in response to systemic cancer stimuli. Unclear are the molecular cues that promote emigration from their endogenous niche and the mechanisms of how they migrate through the source tissue. High resolution two-photon intravital microscopy (IVM) will be instrumental in these efforts. Also, survival of MSCs may be mediated by cell-autonomous and non-cell autonomous mechanisms, including contact with leukocytes or platelets (71). MSCs express several integrins and their ligands, instrumental in binding to selectins, and have been reported to directly interact with leukocytes (72) and platelets (73). However, a major limitation in understanding the recruitment of MSCs has been isolation of viable cells from the circulatory system. Thus, it will be critical to develop methods for capture and analysis of MSCs from blood. Immunoseparation, size separation, and microfluidics may be applied to the reliable isolation of circulating MSCs (74).

MSCs can contribute to several elements of the tumor stroma. The advent of deep-tissue imaging will undoubtedly shed valuable light on how MSCs contribute to the cellular organization of tumors (75). In combination with in vitro and in vivo functional assays, these studies may elucidate contributions of MSCs to tumors and their molecular mechanisms. In addition, we expect that single-cell based approaches will contribute significantly to the understanding of MSC-tumor organization. To fully understand the roles of MSCs in cancer, it will be critical to perform detailed lineage tracing studies of cells derived from exogenous or endogenous MSCs and the effect on the tumor when these progeny are selectively depleted. Because MSC function in tumors is context dependent, it will be critical to evaluate the cause of this dependency by performing RNAi, overexpression, or drug screening for tumor and MSC factors that cause MSCs to promote or inhibit tumor growth in different tumor types.

Furthermore, the mechanisms of MSC homing to (rolling, adhesion, transmigration), survival in, and organization (differentiation and localization) within tumors and normal organs remain to be investigated systematically. Specifically, the question of to what extent selective interactions of MSCs with tumor blood vessels versus other blood vessels contributes to homing remains open, and may be answered with advent of IVM (75). In addition, questions remain as to the ability of MSCs to home to pre-metastatic (58), micrometastatic and macrometastatic growing and dormant (e.g., in the bone) niches (76). Indeed, many of these steps cannot be recapitulated with tail-vein injections of tumor cells and require more complex animal models. Moreover, the ability of MSCs to home to metastases other than in the lung and liver remains to be established. Understanding if and how MSCs cross the blood-brain barrier for treatment of brain metastases will be critical. Finally do MSCs home more efficiently to these metastases, are they preferentially retained there, and/or does the niche promote their proliferation or survival?

Also, MSC spatiotemporally organize themselves in tumors, contributing to different microenvironments, and differentiating into various tumor stroma cells. The significance and the mechanisms of this organization are unclear. Previous approaches of understanding cell fate and environment have been limited by low spatial and/or temporal resolution, required genetic modifications of cells, and/or could not detect multiple molecules. Today, high-resolution two-photon IVM (75), coupled with spatiotemporal FRET-based aptamer microenvironment sensors (SMS; (77); Figure 5) can be used. Such studies would allow probing the spatiotemporal localization of MSCs and measuring the status of critical signaling molecules or events. By coupling these approaches with gain and loss of function studies, it will be possible to directly study the interactions between MSCs and tumors in vivo.

In addition, MSCs have tremendous therapeutic potential due to their immunomodulation, tumor homing and integration capacity, and ability to carry tumoricidal agents. To exploit this for therapy, it is critical to further develop non-genetically engineered MSCs for localized drug delivery. Moreover, MSC homing to tumors may be improved and fine-tuned using genetic, enzymatic, and chemical modification of the MSC surface. For example, preformed SLeX or P/L-selectin targeting aptamer moieties can be anchored to the MSC surface. These have been shown to improve homing to inflamed endothelium both in vitro and in vivo (78–81).

An unexplored potential is use of MSCs in combinatorial therapies, such as incorporating MSC-based therapies with traditional drug therapies, delivery of multiple drugs with one MSC preparation, or modification of both MSC homing and drug loading (Figure 5). As the regulation of cancer cell pathways and signaling by MSCs become better understood, it may be possible to target cancers through a combination of directed therapies and MSCs. Due to the tight association between cancer stem cells and MSCs, targeting the CSC population or niche with MSCs is also an interesting, unexplored direction.

Finally, therapies to modulate the mechanisms of endogenous MSC-tumor interaction have great potential. For example, therapies similar to those aimed at limiting leukocyte homing in inflammation, may also have utility in attenuating MSC homing to tumors. For instance, since TNF-α induced adhesion of MSCs to endothelial cells, anti-inflammatory drugs such as parthenolide could decrease MSC accumulation in tumors (82). Clinically, the utility of blocking MSC homing to tumors will clearly be context and diagnosis-dependent. In the end, these studies will shape our understanding of how tumors integrate with and exploit the biology of the whole organism.

We strongly believe that understanding how both endogenous and exogenous MSCs interact with cancer cells, both in the context of cancer biology and therapeutics, will have enormous benefits in the future. Developing a solid mechanistic and biological understanding of MSCs in cancer will lead to not only improved MSC-based therapies, but open up new areas of research and unveil biology not previously appreciated.

## Supplementary Tables




