# Supplementary material for: To grab the stroma by the horns: From biology to cancer therapy with mesenchymal stem cells
Source: Oncotarget. 2013 May 31;4(5):651–64. doi: 10.18632/oncotarget.1040 (PMC3742827; doi:10.18632/oncotarget.1040)
Supplement: Supplementary file 1 [file oncotarget-04-651-s001.docx]

To grab the stroma by the horns: From biology to cancer therapy with mesenchymal stem cells – Droujinine et al

**Supplementary Table 1: Evidence of MSC homing to tumors**

| **Cancer Model** | **MSC Source** | **Observations** | **Reference** |
| --- | --- | --- | --- |
| Autochthonous pancreatic insuloma | GFP bone marrow transplantation (BMT) | 25% of myofibroblasts were bone marrow derived; clustered on edge of tumor | (Direkze et al., 2004) |
| Pancreatic cancer cell xenograft | GFP BMT | Bone marrow contributed significantly to tumor endothelial and myofibroblast cell populations and increased with tumor progression | (Ishii et al., 2003) |
| Mouse ovarian tumor graft | GFP BMT | Bone marrow-derived cells contributed significantly to the FSP+ and FAP+ components of the stroma | (Kidd et al., 2012) |
| Mouse breast tumor graft | GFP adipose tissue transplant | Adipose-derived cells contributed significantly to the α-SMA and NG2 components of the stroma | (Kidd et al., 2012) |
| Inflammatory model of gastric cancer | GFP BMT | 20% of CAFs are derived from the bone marrow and promote tumor growth; recruitment to the tumor is TGF-β- and SDF-1α-dependent | (Quante et al., 2011) |
| Colon cancer xenograft | Exogenous adipose-tissue derived MSCs | Specifically and robustly incorporated into melanoma xenografts | (Kucerova et al., 2008) |
| Melanoma xenograft | Exogenous bone marrow-derived MSCs | MSCs incorporated into experimental xenografts and metastases and proliferated | (Studeny et al., 2002) |
| Glioma xenograft | Exogenous bone marrow-derived MSCs | Intra-arterial delivery of MSCs led to specific engraftment at gliomas | (Yong et al., 2009) |
| Xenogeneic and syngeneic breast carcinomas | Exogenous luciferase+ bone marrow-derived MSCs | Robust and specific recruitment to tumors, as measured with bioluminescent imaging | (Kidd et al., 2009) |
| Mouse breast tumor graft | Exogenous luciferase+ bone marrow-derived MSCs | Recruitment of MSCs was increased following tumor irradiation, as measured with bioluminescent imaging | (Klopp et al., 2007) |
| Mouse breast tumor graft and experimental metastasis | Exogenous luciferase-GFP bone marrow-derived MSCs | MSCs were recruited to both primary tumors and metastases; MSCs in the primary tumor underwent an osteogenic differentiation, while those in the lungs underwent an adipogenic differentiation | (Wang et al., 2009) |
| Kaposi’s sarcoma xenograft | Exogenous bone marrow-derived MSCs labeled with superparamagnetic iron oxide particles | MSCs robustly and specifically homed to Kaposi’s sarcoma tumor cells, as assayed with MRI following intravenous administration | (Khakoo et al., 2006) |
| Colon cancer xenograft | Exogenous HSV1-TK positive bone marrow-derived MSCs | Robust and specific engraftment of MSCs, including to microscopic tumors, as measured with positron emission tomography following intravenous injection | (Hung et al., 2005) |
| Breast tumor xenograft | Exogenous bone marrow-derived MSCs | MSCs localized to both primary tumors and metastases following intravenous injection | (Loebinger et al., 2009) |
| Melanoma lung metastasis model | Exogenous bone marrow-derived MSCs | MSCs localized to disseminated tumor cells in an experimental model of lung metastasis of melanoma following intravenous delivery | (Ren et al., 2008a) |
| Prostate cancer lung metastasis model | Exogenous bone marrow-derived MSCs | MSCs localized to disseminated prostate cancer cells, in an experimental model of lung metastasis following intravenous administration | (Ren et al., 2008b) |
| Breast and melanoma metastasis xenograft model | Exogenous bone marrow-derived MSCs | MSCs localized and expanded in multiple models of experimental metastasis in vivo following intravenous delivery | (Studeny et al., 2004) |
| Rat model of glioma | Exogenous bone marrow-derived MSCs | Intratumoral, but not intravenous, delivery of MSCs led to robust and specific engraftment in the tumor along the vasculature | (Bexell et al., 2009) |
| Ovarian tumor xenograft | Exogenous bone marrow-derived MSCs | MSCs specifically localized to tumor xenografts within the peritoneum following intraperitoneal injection | (Mader et al., 2009) |
| Breast tumor xenograft | Exogenous adipose-derived MSCs | Adipose-derived MSCs robustly and specifically incorporated into the xenograft following intravenous administration | (Grisendi et al., 2010) |

**Supplementary Table 2: Summary of evidence for MSC organization within tumors**

| Model | Finding | Reference |
| --- | --- | --- |
| Exogenous MSCs in intracranial gliomas after intravenous administration | -MSC derived cells initially cluster near blood vessels, subsequently heterogeneously dispersing throughout the tumor mass in 3 to 4 days | (Yong et al., 2009) |
| Nude athymic mice with intraperitoneal human ovarian cancers locally injected with exogenous adipose-derived MSCs | -MSCs specifically infiltrated the tumor nodules on their surface and parenchyma 24 h after injection, but only minimally localized to normal tissues | (Mader et al., 2009) |
| Exogenous adipose-derived human MSC integration to tumors | -MSCs present as single cells and clusters, strings, and individual cells, of unknown differentiation status | (Grisendi et al., 2010) |
| Exogenous human MSC in pre-established MDA-MB-231 lung metastases | -MSCs localize as small patches or single cells within metastases and the surrounding lung parenchyma | ( Loebinger et al., 2009) |
| Exogenous MSC organization in metastases, subcutaneous tumors, and peritoneal tumors (xenogeneic and syngeneic breast carcinomas) | -MSCs present as strings, patches or single cells throughout the tumors | (Kidd et al., 2009) |
| Exogenous MSC reorganization after tumor irradiation (mouse breast tumor graft) | -MSCs were mostly associated with blood vessels in non-irradiated tumors; irradiation caused MSCs to become more localized to the tumor parenchyma | (Klopp et al., 2007) |
| Exogenous MSC-tumor coinjection (intravenous), MSC intravenous injection after tumor establishment, MSC localization to subcutaneous tumors | -intravenous coinjection of mouse MSCs and tumor cells resulted in MSCs being well dispersed within lung metastases 11 days following injection.  -MSC injection to mice with pre-established metastases resulted in MSC clustering immediately adjacent to and within tumors in patches  -MSC-derived cells localized to lung metastases underwent osteogenic differentiation, while MSCs localized to subcutaneous tumors adopted an adipogenic fate  - the lung with tumor cells contained less of osteoblastic differentiation inhibitor TGFβ1, increased levels of alkaline phosphatase (ALP) and osteocalcin and the osteoblastic transcription factor RUNX2-2 than the normal lung or cultured tumor cells  -subcutaneous breast tumors had less adipocyte inhibitory but osteoblast promoting BMP-2 than lungs or cultured breast cancer cells, and less ALP and osteocalcin than lung with tumors | (Wang et al., 2009) |
| Labeled endogenous bone marrow cell fate tracking experiments | -A significant proportion of tumor associated myofibroblasts and fibroblasts originate from the bone marrow (cell fate tracking experiments) | (Direkze et al., 2004; Ishii et al., 2003) |
| Colon cancer xenograft; Exogenous HSV1-TK positive bone marrow derived human MSCs | -MSCs contributed to capillary and larger vessel endothelial cells | (Hung et al., 2005) |
| Inflammatory model of gastric cancer; labeled endogenous bone marrow transplant | -Bone marrow contributes to 20% of tumor associated myofibroblasts; MSCs differentiate to myofibroblasts in vitro  - MSCs and/or MSC derived myofibroblasts were recruited to, survived in, or differentiated in the tumor in a CXCR4 and/or TGFβ-dependent manner  -growth, self-renewal, senescence, and myofibroblast depletion studies: myofibroblasts form the niche for MSCs via reciprocal signaling and crosstalk.  -myofibroblasts express bone morphogenic protein 4 (BMP4), Wnt5a, and IL-6, and MSCs respond to the presence of myofibroblasts by expressing the BMP inhibitor Gremlin-1  -expression of the Wnt inhibitors DKK1 and Shh dependent on co-culture of MSCs with myofibroblasts, suggesting a delicate signaling network between the two cell types.  -MSCs myofibrobasts often found clustered together as groups, or strings(Quante et al., 2011) | (Quante et al., 2011) |
| Mouse breast and ovarian tumor grafts; labeled endogenous MSC transplantation to bone marrow; labeled adipose transplantation | -Bone marrow contributes to 20% of tumor associated myofibroblasts and majority of fibroblasts  -Bone marrow MSCs contributed to tumor fibroblasts, and some perivascular, fibrovascular, and myofibroblast cells  -Local tissues (including adipose) gave rise to almost all endothelial cells and most perivascular, fibrovascular, and myofibroblast cells | (Kidd et al., 2012) |
| Exogenous adipose stromal cells contribution to distal tumors | -recruited ASCs could differentiate to adipocytes and blood-vessel associated pericytes | (Zhang et al., 2012) |
| Exogenous adipose derived stem cell differentiation | adipose-derived stem cells (which could represent an MSC-like population) differentiated to myofibroblasts in response to tumor derived factors.  -factors including IL-7 and TGFβ have been could be involved  -downregulation of peroxisome proliferator-activated receptor γ (PPARγ) and a decrease in stem cell adipogenic capacity | (Chandler et al., 2012) |
| Ovarian tumor cells and exogenous adipose-derived MSCs | -exosomes from ovarian tumor cells induced myofibroblast markers in adipose-derived MSCs, in a TGFβ-dependent manner | (Cho et al., 2011) |
| Exogenous MSC recruitment to cancer stem cell (CSC) niches | -bone marrow injected MSCs homed to the tumor, distributed throughout the stroma mostly as single cells, and closely associated with the putative CSCs (Liu et al., 2011)  -MSC-like cells were found closely associated with CSC-like cells in human breast cancer samples (Liu et al., 2011)  -MSCs could be isolated from human ovarian carcinomas from different patients, and were multipotent, and had higher percentage of cells that were able to form clones *in vitro* more readily than MSCs derived from BM, adipose, or non-diseased ovary tissue, suggesting a higher proportion of stem cells (McLean et al., 2011; Zhau et al., 2011)  -tumor-derived MSCs likely gave rise to osteoblasts and adipocytes within ovarian tumors *in vivo*, at a higher proportion than non-tumor derived MSCs (McLean et al., 2011) | (Liu et al., 2011; McLean et al., 2011; Zhau et al., 2011) |

**Supplementary Table 3: The function of MSCs in tumors**

| Finding | Reference |
| --- | --- |
| ***Initiation and growth of tumors*** | |
| -human tumor associated fibroblasts (TAFs), but not normal fibroblasts promote tumor growth in mice | (Orimo et al., 2005) |
| -MSCs derived from the bone marrow may give rise to tumor myofibroblasts and promote the growth of gastric tumors | (Quante et al., 2011) |
| -distal adipose-tissue derived MSC-like adipose stromal cells could promote the proliferation of tumor cells | (Zhang et al., 2012) |
| -large numbers of MSC promoted the earlier detection of allogeneic tumors in mice after injection, while having no effect on tumor growth in their model | (Djouad et al., 2006) |
| - MSCs secrete anti-apoptotic and pro-proliferative cytokines and growth factors that may act directly on tumor cells (Da Silva Meirelles et al., 2008; Wu et al., 2010).  -the tumor associated fibroblast (TAF) secreted CXCL-12 (SDF-1) directly promotes the growth of CXCR4 expressing tumors in vivo and in vitro (Orimo et al., 2005). A large proportion of MSCs contribute to TAFs (Kidd et al., 2012). | (Da Silva Meirelles et al., 2008; Kidd et al., 2012; Orimo et al., 2005; Wu et al., 2010) |
| -MSCs modulate cancer cell response to stresses (such as chemotherapy) via secretion of omega-3 and oxo family fatty acids  -blocking the synthesis of these fatty acids and limiting the oral intake of fish and algae oils (rich in these fatty acids) decreased chemotherapy resistance | (Roodhart et al., 2011) |
| ***Tumor angiogenesis*** | |
| - MSCs secrete pro-angiogenic molecules | (Da Silva Meirelles et al., 2008) |
| -hMSC-derived cells induced *in vitro* HUVEC sprouting  -3 days after intravenous injection of hMSCs to pancreatic carcinoma-bearing mice, doubling of blood vessel density occurred within tumors  -MSC-secreted VEGF recruited endothelial cells to tumor | (Beckermann et al., 2008) |
| -TAFs secrete SDF-1 which induces migration of endothelial cell precursors, promotes angiogenesis, and facilitates tumor growth | (Orimo et al., 2005) |
| -tumor and MSC-derived factors were induce an increase in VEGF-dependent HUVEC cell migration | (Chandler et al., 2012) |
| -endogenous MSCs derived from distant adipose tissue differentiated to pericytes and associated with blood vessels | (Zhang et al., 2012) |
| ***Tumor metastasis*** | |
| -hMSCs could reversibly promote the metastasis of several cell lines without themselves colonizing the distant sites  -MSCs may enhance cancer cell motility and/or extravasation to secondary sites  -not clear if MSCs could promote intravasation into the circulatory system or survival in the blood  -tumors induced expression of CCL5 (RANTES) in MSCs, which in turn increased tumor migration and metastasis in a paracrine and/or endocrine manner | (Karnoub et al., 2007) |
| -MSC conditioned media promoted neuroblastoma cell migration in vitro, dependent on SDF1-CXCR4 signaling | (Ma et al., 2011) |
| -enhanced metastasis to the lungs and liver following subcutaneous co-injection of MSCs with MBA-MB-231 cells | (Mi et al., 2011) |
| ***Modulation of immune system function*** | |
| -Distant or local MSCs could induce formation of subcutaneous B16 melanomas in immunocompetent C57Bl/6 mice (which otherwise form very few tumors)  -MSCs inhibited proliferation of mouse or human leukocytes and lymphocytes in vitro via secreted factors | (Djouad et al., 2003) |
| -MSC-like cells isolated from human tumor specimens significantly decreased NK cytotoxicity and mononuclear cell and NK proliferation  -NK activation receptors NKp44 and NKp46 were reduced in cancer | (Johann et al., 2010) |
| -MSC-like cells isolated from bone marrow of chronic myeloid leukemia patients were had less anti-proliferative, anti-activation, and pro-apoptotic effects on T cells in vitro | (Xishan et al., 2011) |
| -MSCs in potentiated recruitment of tumor-promoting macrophages in a chemokine receptor 2 (CCR2)-dependent manner | (Ren et al., 2012) |
| ***Positive regulation of cancer stem cell (CSC) function*** | |
| - human bone marrow (Liu et al., 2011)- or tumor-derived (McLean et al., 2011) MSC-like cells enhance the growth of tumors by positively regulating the proliferation and/or self-renewal of ALDH+ CSCs  -MSCs increased the mammosphere formation capacity of tumor cell lines in vitro (in part via BMP signaling; McLean et al., 2011) and the proportion of ALDH+ CSC-like cells in vivo (Liu et al., 2011)  -co-culture of MSCs and cancer cells led to increased secretion of IL-6, IL-8, CXCL1, CXCL5, CXCL6, and CXCL7 which play roles in regulating the self-renewal of CSCs (Liu et al., 2011) | (McLean et al., 2011  Liu et al., 2011) |
| -secretion of IL-1 by tumor cells induces PGE2 secretion by MSCs  -PGE2, in combination with upregulation of cytokines by MSCs, leads to activation of β-catenin signaling in cancer cells and subsequent formation of CSCs | (Li et al., 2012) |
| ***Tumor Progression Inhibition - Genetic and Other Dependencies*** | |
| -Kaposi sarcoma tumors in mice are growth inhibited when in the presence of MSCs, dependent on cell contact via E-cadherin and Akt inhibition | (Khakoo et al., 2006) |
| -while high numbers of MSCs within tumors may promote tumor initiation, a low number of MSCs may have no or inhibitory effects, suggesting complex dosage effects in pro- and anti- initiation pathways | (Djouad et al., 2006) |

**Supplementary Table 4. The use of MSCs for localized drug delivery to tumors**

| Agent | Mechanism | Advantage of Using MSCs | Results | Challenges | References |
| --- | --- | --- | --- | --- | --- |
| Type I interferons | antiproliferative and proapoptotic | -high degree of toxicity when free IFNs are administered systemically  -MSCs can deliver IFNs and locally release them in tumors | -in a lung metastasis model established by intravenously injecting A375SM and MDA-MB-231 cells, it was found that MSC-IFNβ (but not free IFNβ or MSC-IFNβ in sites other than the tumor) accumulate and survive in lung tumors but not in normal tissues, significantly decrease tumor progression, and prolong the survival of tumor-bearing mice  .  -MSC-IFNβ could decrease prostate cancer lung metastases blood vessel density and proliferation, and increase the cytotoxicity of natural killer cells.  -in a B16-F10 melanoma lung metastasis model, decreases in tumor cell proliferation and vascularization were observed when animals were intravenously injected with MSC-IFNα | -may have broad, context dependent, and currently uncharacterized effects on the immune system and on normal tissues while MSCs are in transit to deliver their cargo to the tumor.  -MSCs frequently reside in non-tumor tissues for some time before accumulating in the tumor itself; effects on normal biology not yet fully understood | (Ren et al., 2008a, 2008b; Studeny et al., 2002, 2004b) |
| Interleukin-12 | IL-12 acts on several immune cells including T, natural killer, and natural killer T cells and induces interferon-γ (IFN-γ) | -free IL-12 is toxic when delivered systemically, because it causes a widespread immune response  -MSCs can deliver IL-12 specifically to tumors and release it locally | -mice with renal clear cell carcinoma tumors were intravenously injected with hMSCs expressing IL-12. MSCs specifically homed to tumors, and a dramatic suppression of tumor growth and prolonged survival of mice of at least to 80 days, was seen. This effect was dependent on IFN-γ and natural killer cells  -MSC-IL-12 (but not free IL-12) treatment into the peritoneal cavity can prophylactically protect mice against subsequent peritoneal tumor challenge. Moreover, MSC-IL-12 treatment did not lead to significant toxicity in the recipient mice, as judged by body weight | -immune response is broad, not well defined, and context dependent | (Chen et al., 2006; Gao et al., 2010; Trinchieri, 2003) |
| Chemokines (CX3CL1) | -at least in part by inducing migration and activation of immune cells | -MSCs locally deliver CX3CL1, avoid recruitment of immune cells to normal organs | MSCs (but not fibroblasts) engineered to secrete CX3CL1 could specifically home to lung metastases and decrease the number of melanoma and adenocarcinoma lung metastases and increase the survival of mice. Moreover, MSC-CX3CL1 induced the migration of CD8+ cytotoxic T cells and NK cells to metastases and the tumoricidal effect on metastases was dependent on the presence of these immune cell types | -Long-term effects of MSCs remaining after therapy is unknown | (Xin et al., 2007) |
| Oncolytic viruses | - target replicating tumor cells and cause their death | -MSCs act as delivery vehicles to protect the viruses from neutralization by the body, and minimize the overall viral dose and systemic toxicity  -tropism of MSCs for tumors lead to preferentially accumulation in tumors | MSCs could home to and decrease the growth of ovarian tumors, and increased survival of mice compared to free adenovirus treatment  -MSCs could protect measles oncolytic virus from neutralization by the body’s immune system  -hMSC-virus treatment was significantly less toxic than the virus alone  -MSCs could specifically home to and deliver infective virus to intracranial gliomas, suppress tumor growth, and significanly prolong animal survival to a time period much greater than the duration of the study | -further work needs to be done in improving the specificity of oncolytic adenoviruses in targeting subsets of cancer cells, while leaving normal tissues spared | (Dembinski et al., 2010; Komarova et al., 2006; Kuruppu and Tanabe, 2005; Mader et al., 2009; Yong et al., 2009) |
| Proapoptotic molecules (e.g., TRAIL) | -tumor necrosis factor related apoptosis inducing ligand (TRAIL) is a pro-apoptotic molecule with relatively selective killing of cancer cells  -MSCs were much more resistant to TRAIL-mediated cytotoxicity than tumor cells | -TRAIL short half life in blood and possible systemic toxicity warrants the use of MSCs as delivery vehicles to tumors | -hMSCs expressing TRAIL under the control of a doxycycline inducible promoter homed to lung metastases and induced the apoptosis of tumor cells  -adipose-derived MSCs-TRAIL were also found to significantly reduce tumor burden after intravenous injection  - intracranial administration of glioma cells and hMSCs transduced with a secretable TRAIL resulted in anti-proliferative and pro-apoptotic effects on the glioma cells, decrease in the bioluminescence glioma signal below detectable levels by 6 days and a significant increase in survival  -MSC-TRAIL was able to induce killing of both putative cancer stem cell (CSC) and non-CSC populations in vitro | -MSC persistence in tissues may remain an issue  -Resistance to TRAIL is common in many cancers | (Grisendi et al., 2010; Loebinger et al., 2009, 2010; Sasportas et al., 2009) |
| Prodrug converting enzymes | -One such enzyme is yeast cytosine deaminase-uracil ribosyltransferase fusion (abbreviated as CD). This enzyme can convert 5-fluorocytidine (FC) to the highly toxic 5-fluorouracil (FU)  -sodium iodide symporter (NIS) can be effective in both imaging MSC biodistribution by selective tumor concentration of ^99m^TcO_4_^-^ or iodide-123, and in concentrating iodide-131 for cancer therapy  -herpes simplex thymidine kinase could act as a prodrug converting enzyme for gancyclovir. | -targeted delivery with MSCs | -Intravenously injected hMSC-CD could home to subcutaneous melanomas and significantly inhibit the growth of melanomas or prostate cancer cells  -When the ^99m^TcO_4_^-^ MSC signal was present only in the tumor, iodine-131 was administered. This treatment resulted in significant and similar reductions in tumor growth. Moreover, it is likely that iodide-131 and FU will also eliminate the MSCs, thus minimizing possible side effects    -Intravenously injected MSCs tranduced with this enzyme could efficiently decrease tumor proliferation and significantly extend life span after gancyclovir injection | -prodrug half life and its effective concentration within tumor after intravenous delivery remain poorly known  -prodrug toxicity in other tissues, particularly the lung | (Bak et al., 2010; Cavarretta et al., 2010; Dwyer et al., 2011; Kucerova et al., 2008) |
| Nano and microparticles | -drugs are encapsulated within particles  -particles are taken up by MSCs and delivered to tumors | -no genetic modifications of MSCs  -targeted delivery of anticancer drugs to tumors  -MSC-particles-drugs integrate to and distribute within tumors  -MSCs may be more resistant to some drugs than cancer cells, but will eventually be killed by the drugs | -nanoparticle surface patches were formed through biotinylation of surface proteins, and attachment of avidin-conjugated nanoparticles  - association of poly-lactic acid NPs (and to a lesser amount per cell of the lipid nanocapsule) with MSCs was retained for at least 7 days, and MSCs were viable, able to differentiate to osteoblasts and adipocytes, and were retained in vivo in the brain glioma tumor mass after intratumoral injection. However, the distribution within the tumor was limited with only a few MSCs present.  -in an in vivo glioma intratumoral injection of MSCs, NPs associated with MSCs were found to redistribute better than NPs not associated with MSCs, and both groups showed cell death. | -patches or particles may interfere with other cellular processes critical for MSC anticancer therapy including homing, incorporation, and survival in tumors | (Cheng et al., 2010; Li et al., 2011; Roger et al., 2010) |

**Supplementary References**

Bak, X. Y., Yang, J., & Wang, S. (2010). Baculovirus-transduced bone marrow mesenchymal stem cells for systemic cancer therapy. *Cancer gene therapy*, *17*(10), 721–9. doi:10.1038/cgt.2010.32

Beckermann, B. M., Kallifatidis, G., Groth, A., Frommhold, D., Apel, A., Mattern, J., Salnikov, A. V, et al. (2008). VEGF expression by mesenchymal stem cells contributes to angiogenesis in pancreatic carcinoma. *British journal of cancer*, *99*(4), 622–31. doi:10.1038/sj.bjc.6604508

Bexell, D., Gunnarsson, S., Tormin, A., Darabi, A., Gisselsson, D., Roybon, L., Scheding, S., et al. (2009). Bone marrow multipotent mesenchymal stroma cells act as pericyte-like migratory vehicles in experimental gliomas. *Molecular therapy : the journal of the American Society of Gene Therapy*, *17*(1), 183–90. doi:10.1038/mt.2008.229

Cavarretta, I. T., Altanerova, V., Matuskova, M., Kucerova, L., Culig, Z., & Altaner, C. (2010). Adipose tissue-derived mesenchymal stem cells expressing prodrug-converting enzyme inhibit human prostate tumor growth. *Molecular therapy : the journal of the American Society of Gene Therapy*, *18*(1), 223–31. doi:10.1038/mt.2009.237

Chandler, E. M., Seo, B. R., Califano, J. P., Andresen Eguiluz, R. C., Lee, J. S., Yoon, C. J., Tims, D. T., et al. (2012a). Implanted adipose progenitor cells as physicochemical regulators of breast cancer. *Proceedings of the National Academy of Sciences of the United States of America*, *109*(25), 9786–91. doi:10.1073/pnas.1121160109

Chen, X., Wang, R., Zhao, X., Wei, Y., Hu, M., Wang, Y., Zhang, X., et al. (2006). Prophylaxis against carcinogenesis in three kinds of unestablished tumor models via IL12-gene-engineered MSCs. *Carcinogenesis*, *27*(12), 2434–41. doi:10.1093/carcin/bgl069

Cheng, H., Kastrup, C. J., Ramanathan, R., Siegwart, D. J., Ma, M., Bogatyrev, S. R., Xu, Q., et al. (2010). Nanoparticulate cellular patches for cell-mediated tumoritropic delivery. *ACS nano*, *4*(2), 625–31. doi:10.1021/nn901319y

Cho, J. A., Park, H., Lim, E. H., Kim, K. H., Choi, J. S., Lee, J. H., Shin, J. W., et al. (2011). Exosomes from ovarian cancer cells induce adipose tissue-derived mesenchymal stem cells to acquire the physical and functional characteristics of tumor-supporting myofibroblasts. *Gynecologic oncology*, *123*(2), 379–86. doi:10.1016/j.ygyno.2011.08.005

Da Silva Meirelles, L., Caplan, A. I., & Nardi, N. B. (2008). In search of the in vivo identity of mesenchymal stem cells. *Stem cells (Dayton, Ohio)*, *26*(9), 2287–99.

Dembinski, J. L., Spaeth, E. L., Fueyo, J., Gomez-Manzano, C., Studeny, M., Andreeff, M., & Marini, F. C. (2010). Reduction of nontarget infection and systemic toxicity by targeted delivery of conditionally replicating viruses transported in mesenchymal stem cells. *Cancer gene therapy*, *17*(4), 289–97. doi:10.1038/cgt.2009.67

Direkze, N. C., Hodivala-Dilke, K., Jeffery, R., Hunt, T., Poulsom, R., Oukrif, D., Alison, M. R., et al. (2004). Bone marrow contribution to tumor-associated myofibroblasts and fibroblasts. *Cancer research*, *64*(23), 8492–5. doi:10.1158/0008-5472.CAN-04-1708

Djouad, F., Bony, C., Apparailly, F., Louis-Plence, P., Jorgensen, C., & Noël, D. (2006). Earlier onset of syngeneic tumors in the presence of mesenchymal stem cells. *Transplantation*, *82*(8), 1060–6. doi:10.1097/01.tp.0000236098.13804.0b

Djouad, F., Plence, P., Bony, C., Tropel, P., Apparailly, F., Sany, J., Noël, D., et al. (2003). Immunosuppressive effect of mesenchymal stem cells favors tumor growth in allogeneic animals. *Blood*, *102*(10), 3837–44. doi:10.1182/blood-2003-04-1193

Dwyer, R. M., Ryan, J., Havelin, R. J., Morris, J. C., Miller, B. W., Liu, Z., Flavin, R., et al. (2011). Mesenchymal Stem Cell-mediated delivery of the sodium iodide symporter supports radionuclide imaging and treatment of breast cancer. *Stem cells (Dayton, Ohio)*, *29*(7), 1149–57. doi:10.1002/stem.665

Gao, P., Ding, Q., Wu, Z., Jiang, H., & Fang, Z. (2010). Therapeutic potential of human mesenchymal stem cells producing IL-12 in a mouse xenograft model of renal cell carcinoma. *Cancer letters*, *290*(2), 157–66. doi:10.1016/j.canlet.2009.08.031

Grisendi, G., Bussolari, R., Cafarelli, L., Petak, I., Rasini, V., Veronesi, E., De Santis, G., et al. (2010). Adipose-derived mesenchymal stem cells as stable source of tumor necrosis factor-related apoptosis-inducing ligand delivery for cancer therapy. *Cancer research*, *70*(9), 3718–29. doi:10.1158/0008-5472.CAN-09-1865

Hung, S.-C., Deng, W.-P., Yang, W. K., Liu, R.-S., Lee, C.-C., Su, T.-C., Lin, R.-J., et al. (2005). Mesenchymal stem cell targeting of microscopic tumors and tumor stroma development monitored by noninvasive in vivo positron emission tomography imaging. *Clinical cancer research : an official journal of the American Association for Cancer Research*, *11*(21), 7749–56. doi:10.1158/1078-0432.CCR-05-0876

Ishii, G., Sangai, T., Oda, T., Aoyagi, Y., Hasebe, T., Kanomata, N., Endoh, Y., et al. (2003). Bone-marrow-derived myofibroblasts contribute to the cancer-induced stromal reaction. *Biochemical and biophysical research communications*, *309*(1), 232–40.

Johann, P.-D., Vaegler, M., Gieseke, F., Mang, P., Armeanu-Ebinger, S., Kluba, T., Handgretinger, R., et al. (2010). Tumour stromal cells derived from paediatric malignancies display MSC-like properties and impair NK cell cytotoxicity. *BMC cancer*, *10*, 501. doi:10.1186/1471-2407-10-501

Karnoub, A. E., Dash, A. B., Vo, A. P., Sullivan, A., Brooks, M. W., Bell, G. W., Richardson, A. L., et al. (2007). Mesenchymal stem cells within tumour stroma promote breast cancer metastasis. *Nature*, *449*(7162), 557–63. doi:10.1038/nature06188

Khakoo, A. Y., Pati, S., Anderson, S. A., Reid, W., Elshal, M. F., Rovira, I. I., Nguyen, A. T., et al. (2006). Human mesenchymal stem cells exert potent antitumorigenic effects in a model of Kaposi’s sarcoma. *The Journal of experimental medicine*, *203*(5), 1235–47. doi:10.1084/jem.20051921

Kidd, S., Spaeth, E., Dembinski, J. L., Dietrich, M., Watson, K., Klopp, A., Battula, V. L., et al. (2009). Direct evidence of mesenchymal stem cell tropism for tumor and wounding microenvironments using in vivo bioluminescent imaging. *Stem cells (Dayton, Ohio)*, *27*(10), 2614–23. doi:10.1002/stem.187

Kidd, S., Spaeth, E., Watson, K., Burks, J., Lu, H., Klopp, A., Andreeff, M., et al. (2012). Origins of the tumor microenvironment: quantitative assessment of adipose-derived and bone marrow-derived stroma. *PloS one*, *7*(2), e30563. doi:10.1371/journal.pone.0030563

Klopp, A. H., Spaeth, E. L., Dembinski, J. L., Woodward, W. A., Munshi, A., Meyn, R. E., Cox, J. D., et al. (2007). Tumor irradiation increases the recruitment of circulating mesenchymal stem cells into the tumor microenvironment. *Cancer research*, *67*(24), 11687–95. doi:10.1158/0008-5472.CAN-07-1406

Komarova, S., Kawakami, Y., Stoff-Khalili, M. A., Curiel, D. T., & Pereboeva, L. (2006). Mesenchymal progenitor cells as cellular vehicles for delivery of oncolytic adenoviruses. *Molecular cancer therapeutics*, *5*(3), 755–66. doi:10.1158/1535-7163.MCT-05-0334

Kucerova, L., Matuskova, M., Pastorakova, A., Tyciakova, S., Jakubikova, J., Bohovic, R., Altanerova, V., et al. (2008). Cytosine deaminase expressing human mesenchymal stem cells mediated tumour regression in melanoma bearing mice. *The journal of gene medicine*, *10*(10), 1071–82. doi:10.1002/jgm.1239

Kuruppu, D., & Tanabe, K. K. (2005). Viral oncolysis by herpes simplex virus and other viruses. *Cancer biology & therapy*, *4*(5), 524–31.

Li, L., Guan, Y., Liu, H., Hao, N., Liu, T., Meng, X., Fu, C., et al. (2011). Silica nanorattle-doxorubicin-anchored mesenchymal stem cells for tumor-tropic therapy. *ACS nano*, *5*(9), 7462–70. doi:10.1021/nn202399w

Liu, S., Ginestier, C., Ou, S. J., Clouthier, S. G., Patel, S. H., Monville, F., Korkaya, H., et al. (2011). Breast cancer stem cells are regulated by mesenchymal stem cells through cytokine networks. *Cancer research*, *71*(2), 614–24. doi:10.1158/0008-5472.CAN-10-0538

Loebinger, M R, Sage, E. K., Davies, D., & Janes, S. M. (2010). TRAIL-expressing mesenchymal stem cells kill the putative cancer stem cell population. *British journal of cancer*, *103*(11), 1692–7. doi:10.1038/sj.bjc.6605952

Loebinger, Michael R, Eddaoudi, A., Davies, D., & Janes, S. M. (2009). Mesenchymal stem cell delivery of TRAIL can eliminate metastatic cancer. *Cancer research*, *69*(10), 4134–42.

Loebinger, Michael R, Kyrtatos, P. G., Turmaine, M., Price, A. N., Pankhurst, Q., Lythgoe, M. F., & Janes, S. M. (2009). Magnetic resonance imaging of mesenchymal stem cells homing to pulmonary metastases using biocompatible magnetic nanoparticles. *Cancer research*, *69*(23), 8862–7.

Ma, M., Ye, J. Y., Deng, R., Dee, C. M., & Chan, G. C.-F. (2011). Mesenchymal stromal cells may enhance metastasis of neuroblastoma via SDF-1/CXCR4 and SDF-1/CXCR7 signaling. *Cancer letters*, *312*(1), 1–10. doi:10.1016/j.canlet.2011.06.028

Mader, E. K., Maeyama, Y., Lin, Y., Butler, G. W., Russell, H. M., Galanis, E., Russell, S. J., et al. (2009). Mesenchymal stem cell carriers protect oncolytic measles viruses from antibody neutralization in an orthotopic ovarian cancer therapy model. *Clinical cancer research : an official journal of the American Association for Cancer Research*, *15*(23), 7246–55. doi:10.1158/1078-0432.CCR-09-1292

McLean, K., Gong, Y., Choi, Y., Deng, N., Yang, K., Bai, S., Cabrera, L., et al. (2011). Human ovarian carcinoma–associated mesenchymal stem cells regulate cancer stem cells and tumorigenesis via altered BMP production. *The Journal of clinical investigation*, *121*(8), 3206–19. doi:10.1172/JCI45273

Mi, Z., Bhattacharya, S. D., Kim, V. M., Guo, H., Talbot, L. J., & Kuo, P. C. (2011). Osteopontin promotes CCL5-mesenchymal stromal cell-mediated breast cancer metastasis. *Carcinogenesis*, *32*(4), 477–87. doi:10.1093/carcin/bgr009

Orimo, A., Gupta, P. B., Sgroi, D. C., Arenzana-Seisdedos, F., Delaunay, T., Naeem, R., Carey, V. J., et al. (2005). Stromal fibroblasts present in invasive human breast carcinomas promote tumor growth and angiogenesis through elevated SDF-1/CXCL12 secretion. *Cell*, *121*(3), 335–48. doi:10.1016/j.cell.2005.02.034

Quante, M., Tu, S. P., Tomita, H., Gonda, T., Wang, S. S. W., Takashi, S., Baik, G. H., et al. (2011). Bone marrow-derived myofibroblasts contribute to the mesenchymal stem cell niche and promote tumor growth. *Cancer cell*, *19*(2), 257–72. doi:10.1016/j.ccr.2011.01.020

Ren, C, Kumar, S., Chanda, D., Kallman, L., Chen, J., Mountz, J. D., & Ponnazhagan, S. (2008). Cancer gene therapy using mesenchymal stem cells expressing interferon-beta in a mouse prostate cancer lung metastasis model. *Gene therapy*, *15*(21), 1446–53. doi:10.1038/gt.2008.101

Ren, Changchun, Kumar, S., Chanda, D., Chen, J., Mountz, J. D., & Ponnazhagan, S. (2008). Therapeutic potential of mesenchymal stem cells producing interferon-alpha in a mouse melanoma lung metastasis model. *Stem cells (Dayton, Ohio)*, *26*(9), 2332–8. doi:10.1634/stemcells.2008-0084

Ren, G., Zhao, X., Wang, Y., Zhang, X., Chen, X., Xu, C., Yuan, Z., et al. (2012). CCR2-dependent recruitment of macrophages by tumor-educated mesenchymal stromal cells promotes tumor development and is mimicked by TNFα. *Cell stem cell*, *11*(6), 812–24. doi:10.1016/j.stem.2012.08.013

Roger, M., Clavreul, A., Venier-Julienne, M.-C., Passirani, C., Sindji, L., Schiller, P., Montero-Menei, C., et al. (2010). Mesenchymal stem cells as cellular vehicles for delivery of nanoparticles to brain tumors. *Biomaterials*, *31*(32), 8393–401. doi:10.1016/j.biomaterials.2010.07.048

Roodhart, J. M. L., Daenen, L. G. M., Stigter, E. C. A., Prins, H.-J., Gerrits, J., Houthuijzen, J. M., Gerritsen, M. G., et al. (2011). Mesenchymal stem cells induce resistance to chemotherapy through the release of platinum-induced fatty acids. *Cancer cell*, *20*(3), 370–83. doi:10.1016/j.ccr.2011.08.010

Sasportas, L. S., Kasmieh, R., Wakimoto, H., Hingtgen, S., Van de Water, J. A. J. M., Mohapatra, G., Figueiredo, J. L., et al. (2009). Assessment of therapeutic efficacy and fate of engineered human mesenchymal stem cells for cancer therapy. *Proceedings of the National Academy of Sciences of the United States of America*, *106*(12), 4822–7. doi:10.1073/pnas.0806647106

Studeny, M., Marini, F. C., Champlin, R. E., Zompetta, C., Fidler, I. J., & Andreeff, M. (2002). Bone marrow-derived mesenchymal stem cells as vehicles for interferon-beta delivery into tumors. *Cancer research*, *62*(13), 3603–8.

Studeny, M., Marini, F. C., Dembinski, J. L., Zompetta, C., Cabreira-Hansen, M., Bekele, B. N., Champlin, R. E., et al. (2004). Mesenchymal stem cells: potential precursors for tumor stroma and targeted-delivery vehicles for anticancer agents. *Journal of the National Cancer Institute*, *96*(21), 1593–603.

Trinchieri, G. (2003). Interleukin-12 and the regulation of innate resistance and adaptive immunity. *Nature reviews. Immunology*, *3*(2), 133–46. doi:10.1038/nri1001

Wang, H., Cao, F., De, A., Cao, Y., Contag, C., Gambhir, S. S., Wu, J. C., et al. (2009). Trafficking mesenchymal stem cell engraftment and differentiation in tumor-bearing mice by bioluminescence imaging. *Stem cells (Dayton, Ohio)*, *27*(7), 1548–58.

Wu, Y., Zhao, R. C. H., & Tredget, E. E. (2010). Concise review: bone marrow-derived stem/progenitor cells in cutaneous repair and regeneration. *Stem cells (Dayton, Ohio)*, *28*(5), 905–15. doi:10.1002/stem.420

Xin, H., Kanehira, M., Mizuguchi, H., Hayakawa, T., Kikuchi, T., Nukiwa, T., & Saijo, Y. (2007). Targeted delivery of CX3CL1 to multiple lung tumors by mesenchymal stem cells. *Stem cells (Dayton, Ohio)*, *25*(7), 1618–26. doi:10.1634/stemcells.2006-0461

Xishan, Z., Guangyu, A., Yuguang, S., & Hongmei, Z. (2011). The research on the immuno-modulatory defect of mesenchymal stem cell from Chronic Myeloid Leukemia patients. *Journal of experimental & clinical cancer research : CR*, *30*, 47. doi:10.1186/1756-9966-30-47

Yong, R. L., Shinojima, N., Fueyo, J., Gumin, J., Vecil, G. G., Marini, F. C., Bogler, O., et al. (2009). Human bone marrow-derived mesenchymal stem cells for intravascular delivery of oncolytic adenovirus Delta24-RGD to human gliomas. *Cancer research*, *69*(23), 8932–40. doi:10.1158/0008-5472.CAN-08-3873

Zhang, Y., Daquinag, A. C., Amaya-Manzanares, F., Sirin, O., Tseng, C., & Kolonin, M. G. (2012). Stromal progenitor cells from endogenous adipose tissue contribute to pericytes and adipocytes that populate the tumor microenvironment. *Cancer research*, *72*(20), 5198–208. doi:10.1158/0008-5472.CAN-12-0294

Zhau, H. E., He, H., Wang, C. Y., Zayzafoon, M., Morrissey, C., Vessella, R. L., Marshall, F. F., et al. (2011). Human prostate cancer harbors the stem cell properties of bone marrow mesenchymal stem cells. *Clinical cancer research : an official journal of the American Association for Cancer Research*, *17*(8), 2159–69. doi:10.1158/1078-0432.CCR-10-2523
