# Supplementary figures and images for: To grab the stroma by the horns: From biology to cancer therapy with mesenchymal stem cells
Source: Oncotarget. 2013 May 31;4(5):651–64. doi: 10.18632/oncotarget.1040 (PMC3742827; doi:10.18632/oncotarget.1040)

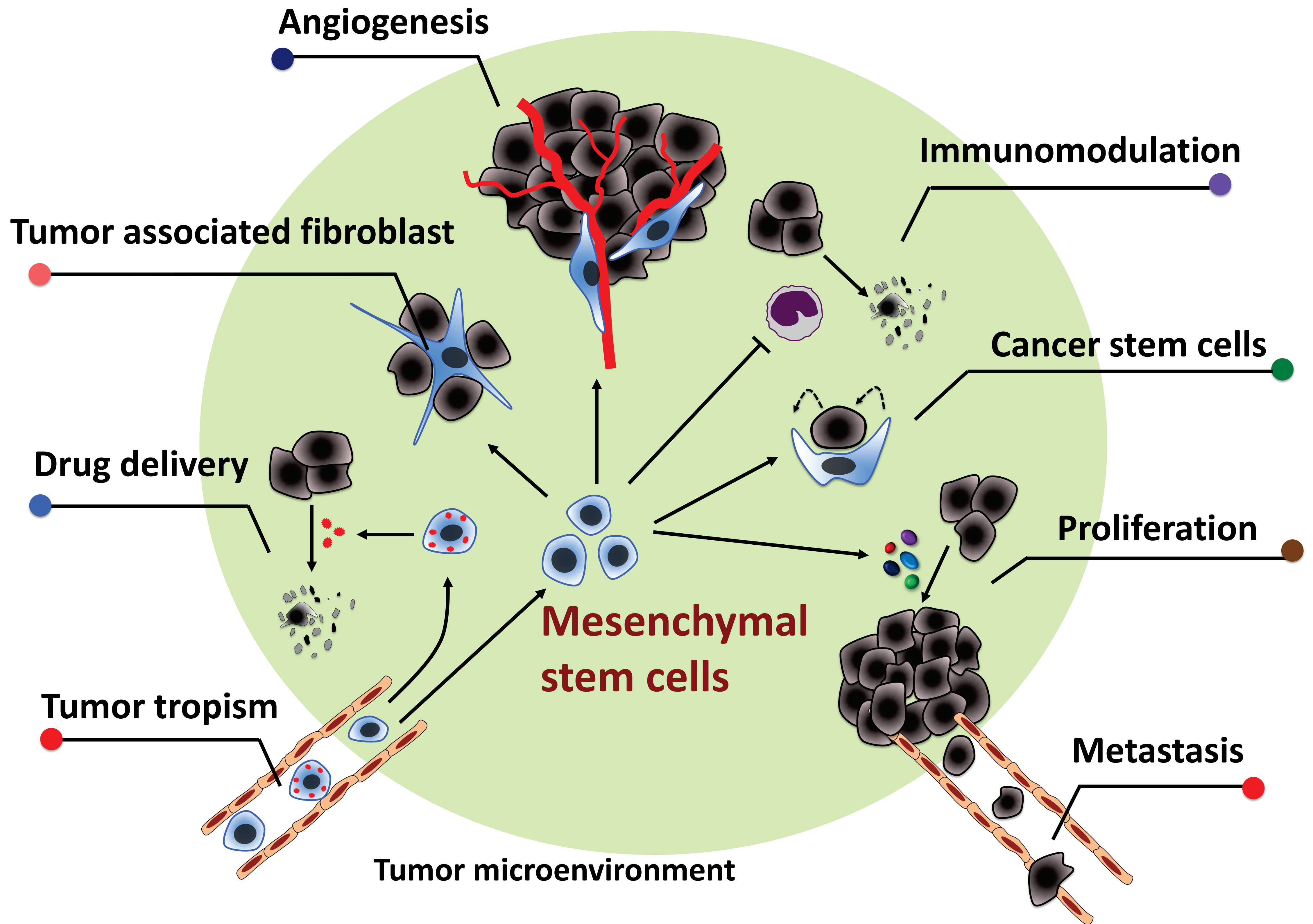

Supplement: Supplementary file 2 [file oncotarget-04-651-s002.pdf]
